# Neurobiological Relationships Between Neurodevelopmental Disorders and Mood Disorders

**DOI:** 10.3390/brainsci15030307

**Published:** 2025-03-14

**Authors:** Amanda Gollo Bertollo, Camila Ferreira Puntel, Brunna Varela da Silva, Marcio Martins, Margarete Dulce Bagatini, Zuleide Maria Ignácio

**Affiliations:** 1 Postgraduate Program in Neuroscience, Federal University of Santa Catarina (UFSC), Florianópolis 88040-900, SC, Brazil; amandagollo@gmail.com; 2 Laboratory of Physiology, Pharmacology and Psychopathology, Federal University of Fronteira Sul (UFFS), Chapecó 89815-899, SC, Brazil; camila.puntel@estudante.uffs.edu.br (C.F.P.); brunnavbiomed@gmail.com (B.V.d.S.); 3 Postgraduate Program in Contemporary Cultural Studies, Federal University of Mato Grosso (UFMT), Cuiabá 78060-900, MT, Brazil; falandodamentehumana@gmail.com; 4 Cell Culture Laboratory, Graduate Program in Biomedical Sciences, Federal University of Fronteira Sul (UFFS), Chapecó 89815-899, SC, Brazil; margaretebagatini@gmail.com

**Keywords:** neurodevelopmental disorders, mood disorders, biological mechanisms, synaptic impairments, attention-deficit/hyperactivity disorder, autism spectrum disorder, intellectual disability, learning disorders, communication disorders

## Abstract

According to the Diagnostic and Statistical Manual of Mental Disorders (DSM-5), neurodevelopmental disorders (NDDs) are a group of conditions that arise early in development and are characterized by deficits in personal, social, academic, or occupational functioning. These disorders frequently co-occur and include conditions such as autism spectrum disorder (ASD) and attention-deficit/hyperactivity disorder (ADHD). Mood disorders (MDs), such as major depressive disorder and bipolar disorder, also pose significant global health challenges due to their high prevalence and substantial impact on quality of life. Emerging evidence highlights overlapping neurobiological mechanisms between NDDs and MDs, including shared genetic susceptibilities, neurotransmitter dysregulation (e.g., dopaminergic and serotonergic pathways), neuroinflammation, and hypothalamic–pituitary–adrenal (HPA) axis dysfunction. Environmental factors such as early-life adversity further exacerbate these vulnerabilities, contributing to the complexity of their clinical presentation and comorbidity. Functional neuroimaging studies reveal altered connectivity in brain regions critical for emotional regulation and executive function, such as the prefrontal cortex and amygdala, across these disorders. Despite these advances, integrative diagnostic frameworks and targeted therapeutic strategies remain underexplored, limiting effective intervention. This review synthesizes current knowledge on the shared neurobiological underpinnings of NDDs and MDs, emphasizing the need for multidisciplinary research, including genetic, pharmacological, and psychological approaches, for unified diagnosis and treatment. Addressing these intersections can improve clinical outcomes and enhance the quality of life for individuals affected by these disorders.

## 1. Introduction

Neurodevelopmental disorders (NDDs) like autism spectrum disorder (ASD), attention-deficit/hyperactivity disorder (ADHD), and mood disorders (MDs), such as major depressive disorder (MDD) and bipolar disorder, present significant public health challenges. These conditions are prevalent worldwide, with NDDs affecting a significant portion of children and adolescents. ADHD affects around 8.5%, ASD approximately 2.9%, and specific learning disorders about 6.4%, often accompanied by comorbid psychiatric conditions like anxiety and depression [1,2]. Additionally, 18% of individuals with NDDs exhibit genetic alterations associated with developmental delays and ASD, emphasizing the need for early diagnosis [3]. Common mental disorders like anxiety and depression affect 17.6% of the population annually, with higher prevalence among women and individuals with lower socioeconomic status [4].

The clinical presentations of NDDs and MDs frequently overlap, with symptoms like emotional dysregulation, impaired executive function, and social difficulties common to both. Studies show that individuals with ADHD often experience emotional instability resembling depressive or bipolar symptoms, while those with ASD may exhibit heightened anxiety and depression, suggesting shared mechanisms [5,6]. Alterations in brain regions involved in emotional regulation, like the prefrontal cortex and amygdala, contribute to these overlapping symptoms [7].

Neurobiological mechanisms common to both NDDs and MDs include disruptions in dopaminergic and serotonergic systems, neuroinflammation, and genetic vulnerabilities. The dopamine D1 receptor plays a key role in regulating neuroinflammation, and variations in the serotonergic system, like in the HTR1A gene, contribute to psychiatric and motor complications [8,9]. Brain imaging studies reveal functional alterations in areas like the prefrontal cortex and amygdala, reinforcing the overlap between these disorders [10].

Environmental factors, particularly childhood trauma, interact with genetic predispositions to influence the onset and severity of both NDDs and MDs. Exposure to trauma is linked to increased risks of MDs in children of bipolar patients [11], and genetic variations in the CRHR1 gene, combined with childhood stress, increase the risk of suicidal behaviors in individuals with affective disorders [12]. These experiences exacerbate neurobiological vulnerabilities, such as dysregulated stress response systems [13].

Despite advances in understanding NDDs and MDs individually, the shared neurobiological processes between them remain underexplored, hindering the development of effective diagnostic and therapeutic strategies. Current diagnostic frameworks often treat comorbidities separately, leading to fragmented care [5]. Disparities in diagnosis and management, such as those related to socioeconomic status, worsen outcomes for individuals with co-occurring disorders [14]. A shift toward interdisciplinary research is essential, combining psychiatry, neurology, and developmental neuroscience to address the complexity of these disorders [15].

This article aims to review the shared neurobiological mechanisms of NDDs and MDs, focusing on specific disorders, including ASD, ADHD, and intellectual disabilities. By identifying common pathophysiological mechanisms, this work seeks to inform future research and clinical practices to improve outcomes for individuals affected by these complex and often co-occurring conditions.

## 2. Common Neurobiological Mechanisms Between Neurodevelopmental Disorders and Mood Disorders

**Figure 1:**
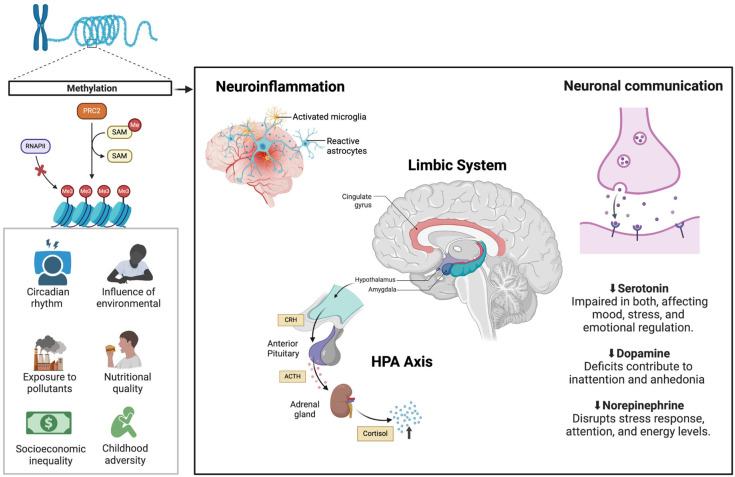
Common neurobiological pathways in NDDs and MDs. Environmental factors influence DNA methylation, where PRC2 (Polycomb Repressive Complex 2) regulates gene expression by adding Me (methyl groups) via SAM (S-adenosylmethionine), inhibiting RNAPII (RNA Polymerase II) and altering neural function. These changes contribute to neuroinflammation (activated microglia, reactive astrocytes) and HPA axis (hypothalamic–pituitary–adrenal) dysfunction, increasing CRH (Corticotropin-Releasing Hormone), ACTH (Adrenocorticotropic Hormone), and cortisol release. Alterations in the limbic system, including the cingulate gyrus, amygdala, and hypothalamus, impact emotional regulation. Neurotransmitter dysregulation, with reduced serotonin, dopamine, and norepinephrine, impairs mood, attention, and stress response, reinforcing the shared pathophysiology of NDDs and MDs.

Figure 1 illustrates some common neurobiological mechanisms between neurodevelopmental disorders and mood disorders.

From the early years of life, NDDs, such as ASDs and ADHDs, significantly impact cognitive and behavioral trajectories, supported by robust genetic and neurobiological foundations. These conditions often overlap with MDs, such as MDD and bipolar disorder, characterized by persistent emotional changes and dysfunctions in neurotransmitter systems [16]. Studies show that NDDs and MDs exhibit common alterations in neural circuits, such as the limbic system and the prefrontal cortex, critical areas for emotional and behavioral regulation [17]. Environmental factors, such as early stress, can also exacerbate manifestations in both categories [18].

Evidence suggests that NDDs and MDs are not distinct categories but share complex neurobiological foundations. Alterations in mechanisms such as synaptic plasticity, neurotransmitter metabolism, and neural connectivity contribute to developing symptoms in both conditions. Identifying these common points is essential for advancing clinical understanding, as MD symptoms often mask or exacerbate NDD manifestations [19]. For instance, genetic studies highlight the overlap of variants affecting brain development and neurogenesis genes, indicating biological connections between these disorders [18].

Identifying shared neurobiological mechanisms between NDDs and MDs is crucial to enhancing diagnostic precision and therapeutic interventions. Both types of disorders exhibit alterations in the HPA axis, which regulates stress responses, and dysfunctions in neurotransmitters such as dopamine and serotonin, directly influencing emotional and behavioral processes [17,18]. Studies suggest that these changes can be observed in functional brain imaging, revealing abnormal connectivity patterns in NDDs and MDs [16]. Moreover, neuroinflammation, mediated by immune system alterations, is a common mechanism that aggravates symptoms in both conditions [19].

The overlap of these mechanisms has significant clinical implications. For example, identifying common biological markers can guide the development of targeted therapies that address multiple symptoms simultaneously, such as neurotransmitter modulators or interventions to regulate the HPA axis [18]. Additionally, personalized treatment strategies based on shared mechanisms can prevent the progression of comorbidities and improve patients’ quality of life [17].

This neurobiological overlap is also reflected in diagnostic and therapeutic challenges. For example, MD symptoms can hinder the identification of core ASD characteristics. Recent studies highlight the importance of careful differentiation between these conditions to avoid misdiagnoses. According to Wittkopf et al. [20], a comprehensive evaluation that includes emotional and behavioral dimensions is crucial, as proper clinical management depends on accurately identifying the disorders involved.

MDs, such as MDD and bipolar disorder, are characterized by persistent emotional state changes, ranging from episodes of deep sadness to extreme euphoria. These disorders have a complex neurobiological basis, influenced by genetic factors, neurotransmitter alterations, and dysfunctions in brain circuits. Studies show that imbalances in serotonin, dopamine, and norepinephrine systems play central roles in developing and maintaining MD symptoms, directly affecting mood regulation and stress response [16]. Additionally, alterations in the prefrontal cortex, responsible for decision-making and emotional control, and the limbic system, such as the amygdala and hippocampus, are strongly associated with dysfunctional emotional patterns [17].

Genetic factors also significantly contribute to the predisposition to MDs. Polymorphisms in genes related to serotonin transporters and dopamine receptors are frequently identified in individuals with depression and bipolar disorder [18]. These genetic changes, combined with environmental factors such as childhood trauma, can modify synaptic plasticity and neurogenesis, resulting in vulnerabilities to developing these disorders [19].

One main point of convergence between NDDs and MDDs is genetic overlap. Studies reveal that variants in genes associated with brain development regulation, such as those involved in synaptic plasticity and neurotransmitter transport, are shared between NDDs and MDs [16]. These genetic similarities suggest that both disorders may derive from underlying neurobiological processes related to neural development.

Structural and functional brain changes are also commonly observed. For example, reduced hippocampal volume and dysfunctions in prefrontal cortex connectivity with subcortical areas are reported in individuals with NDDs and MDs [18]. These changes affect emotional and behavioral regulation, contributing to shared symptoms such as impulsivity, emotional dysfunction, and attention deficits.

Another common mechanism is the dysfunction of the HPA axis, which regulates the stress response. HPA axis alterations lead to abnormal cortisol levels, which can negatively impact brain plasticity and exacerbate symptoms of both disorders [17]. Moreover, chronic stress and neuroinflammation, associated with immune system imbalances, are frequently observed in individuals with NDDs and MDs, suggesting a link between stress responses and shared pathological mechanisms [19].

## 3. Autism Spectrum Disorder

**Figure 2:**
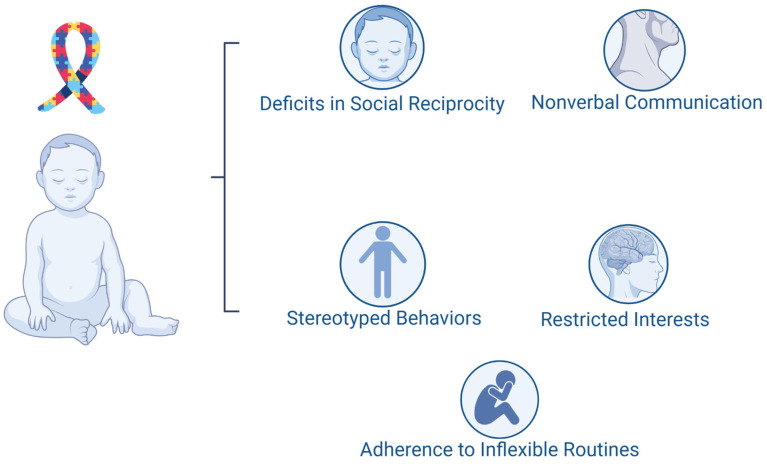
Core diagnostic criteria for autism spectrum disorder (ASD). ASD diagnosis is based on impairments in two main domains: (1) social communication deficits, including difficulties in social reciprocity and nonverbal communication, and (2) restricted and repetitive behaviors, encompassing stereotyped behaviors, restricted interests, and adherence to inflexible routines. These characteristics contribute to the heterogeneity of ASD presentations and impact daily functioning across different developmental stages.

ASD is a neurodevelopmental condition characterized by persistent impairments in communication and social interaction, in addition to restricted and repetitive patterns of behavior, interests, or activities, with symptoms present since childhood that impact daily functioning. According to the fifth edition of the DSM-5 [21], as illustrated in Figure 2, the diagnosis of ASD is based on manifestations observed in two main domains: (1) deficits in social reciprocity and nonverbal communication and (2) stereotyped behaviors, restricted interests, and adherence to inflexible routines [22]. The diagnostic complexity of ASD arises from its broad phenotypic variability, with varying degrees of severity and associated manifestations, including psychiatric comorbidities and cognitive alterations. This variability is linked to genetic and environmental factors influencing neurodevelopment from the earliest stages of life [23].

The main signs and symptoms of ASD include deficits in social communication and restrictive and repetitive behaviors, which vary in severity and functional impact among individuals. Difficulties in social communication manifest as challenges in initiating or maintaining interactions, understanding implicit rules of reciprocity, and adjusting behavior to different social contexts. Individuals with ASD often show limited use of gestures and facial expressions, hindering the conveyance of intentions and emotions and compromising the formation of interpersonal relationships [23]. Furthermore, emotional regulation and the presence of alexithymia—difficulty in identifying and expressing emotions—have been identified as critical factors for understanding the emotional impact of ASD, especially in high-functioning cases [24].

From a biological perspective, ASD is associated with complex and multifactorial alterations involving genetic and neurological mechanisms. Genome-wide association studies (GWASs) have identified genetic variants in genes related to synaptogenesis, neuronal plasticity, and the regulation of neural development, such as SH3 and multiple ankyrin repeat domains 3 (SHANK3), neurexin 1 (NRXN1), and chromodomain helicase DNA binding protein 8 (CHD8) [25].

In addition to these well-established variants, recent studies have highlighted other genes with potential roles in ASD and comorbid NDDs. For instance, mutations in the E3 ubiquitin protein ligase encoded by the ARIH2 gene, which is involved in post-translational modifications, have been identified in individuals with co-occurring ASD and intellectual disability (ID), suggesting disruption of protein autoinhibition mechanisms critical for neural function [26].

Similarly, deletions affecting the ZDHHC15 gene, a candidate for X-linked forms of intellectual disability and ASD, have been associated with ASD in multiplex families, reinforcing its role in synaptic function and sensory processing alterations with incomplete penetrance and variable expressivity [27]. Moreover, the transient receptor potential channel 6 (TRPC6) gene has emerged as an ASD risk factor within an oligogenic model, with loss-of-function mutations linked to deficits in social behaviors, sleep regulation, and cognitive functions, as evidenced through both human studies and functional models in Drosophila melanogaster [28]. These genetic alterations affect communication between neurons and may impair functional connectivity between different brain regions, particularly in areas associated with socialization and cognition, while contributing to the heterogeneity and frequent comorbidity observed between ASD and other NDDs.

Additionally, changes in white and gray matter volume and functional and structural connectivity are frequently observed in individuals with ASD. Functional neuroimaging analyses indicate hyperconnectivity in local circuits and hypoconnectivity in long-distance networks, particularly those involving the prefrontal cortex, superior temporal cortex, and amygdala [29]. These findings suggest an imbalance in neural network integration, contributing to the disorder’s behavioral and cognitive characteristics.

Restrictive and repetitive behaviors constitute another central aspect of ASD, including the repetition of words or phrases (echolalia), stereotyped motor movements, and inflexible adherence to routines or patterns. Individuals with ASD may also exhibit intense and unusual interests, such as fixation on numbers or specific objects [22,23]. Sensory alterations, such as hypersensitivity or hyposensitivity to environmental stimuli, are common and can exacerbate repetitive behaviors, impacting social interaction and environmental adaptation [23].

Psychiatric comorbidities are common in ASD and include ADHD, anxiety, obsessive-–compulsive disorders (OCDs), and MDs. The presence of these conditions often complicates diagnostic differentiation, as highlighted by Wittkopf et al. [20], who emphasized the importance of carefully evaluating overlapping symptoms to distinguish ASD from mood or anxiety disorders. Emotional challenges such as difficulties in regulating emotions and irritability exacerbate the adaptive and social challenges faced by individuals with ASD and their families.

Standardized tools, such as the Autism Diagnostic Observation Schedule (ADOS-2) and the Autism Diagnostic Interview-Revised (ADI-R), are widely used to assess the severity and specific manifestations of ASD. The ADOS-2 evaluates difficulties in social interaction and repetitive behavioral patterns, while the ADI-R allows for retrospective analysis of early childhood [23]. This integrative approach is essential for addressing the heterogeneity of ASD, providing a more accurate diagnosis and targeted interventions.

The broad phenotypic variability in ASD, which includes differences in intelligence quotient (IQ), adaptive skills, and emotional problems, poses a challenge in both clinical practice and research [23]. Normative models have been proposed to identify homogeneous subgroups, facilitating the personalization of interventions. This perspective makes therapeutic strategies more practical, focusing on adaptive and functional outcomes [23,24].

## 4. Attention-Deficit/Hyperactivity Disorder

**Figure 3:**
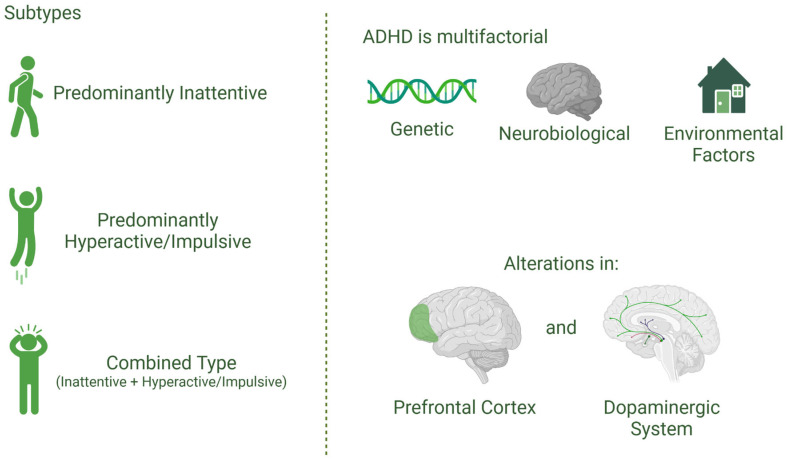
Subtypes and neurobiological basis of attention-deficit/hyperactivity disorder (ADHD). ADHD is classified into three main subtypes: predominantly inattentive, predominantly hyperactive/impulsive, and combined type, which includes characteristics of both. The etiology of ADHD is multifactorial, involving genetic, neurobiological, and environmental factors. Studies indicate that alterations in the prefrontal cortex and the dopaminergic system play a crucial role in the executive function deficits observed in individuals with ADHD.

Figure 3 illustrates the subtypes of ADHD and highlights its multifactorial etiology, as well as the neurobiological alterations associated with the disorder.

ADHD is a neurodevelopmental disorder that has been extensively studied due to its significant impact on individuals’ lives. It is characterized by a persistent pattern of inattention, hyperactivity, and impulsivity, which interferes with functioning and development, manifesting since childhood. According to the criteria established by the DSM-5, symptoms must be present for at least six months, be inappropriate for the individual’s developmental level, and cause significant impairment in two or more settings, such as school, home, or work environments. ADHD is classified into three main subtypes: predominantly inattentive, predominantly hyperactive/impulsive, and combined type, which encompasses characteristics of the previous subtypes. Each subtype presents distinct manifestations, although they often overlap, forming a complex spectrum of symptoms [21].

Among the symptoms related to inattention, difficulties in organizing tasks and activities, frequent memory lapses, and a tendency to lose necessary everyday objects are observed. Hyperactivity symptoms are evident in behaviors such as constant restlessness and difficulty remaining seated for extended periods. Impulsivity manifests in hasty actions, such as interrupting conversations and difficulty waiting one’s turn in group situations. When not adequately managed, these behaviors can lead to additional problems in social interactions, academic performance, and family life [30].

The etiology of ADHD is multifactorial, involving a complex interplay of genetic, epigenetic, neurobiological, and environmental factors. Recent research has highlighted DNA methylation variation, which influences ADHD symptom trajectories, with genes like SKI, ZNF544, ST3GAL3, and PEX2 showing associations with ADHD at birth. These findings underscore the role of epigenetic mechanisms in modulating ADHD symptoms, particularly in neurodevelopmental processes. Gene–environment interactions further shape the disorder’s presentation, pointing to the significance of early-life exposures and genetic expression in ADHD development [31]. Studies point to alterations in the prefrontal cortex and the dopaminergic system as central to the executive control difficulties observed in the disorder. These alterations also contribute to the higher prevalence of comorbidities, such as mood and anxiety disorders, frequently reported in individuals with ADHD and ASD [30].

Among psychiatric comorbidities, MDs, such as depression and anxiety, are significantly prevalent in both ADHD and ASD. Individuals with these conditions often exhibit symptoms of irritability, emotional regulation difficulties, and a pronounced vulnerability to stress. This vulnerability is a key characteristic that contributes to the worsening of their clinical conditions, underscoring the urgent need to address this issue. Studies demonstrate that the association between ASD and MDs reflects a complex interaction between neurobiological alterations, HPA axis dysfunctions, and environmental factors [30,32].

Moreover, the risk of depression and suicidal behaviors in individuals with ASD, especially in high-functioning cases, highlights the need for specific and targeted interventions. According to Ruggieri [32], factors such as social isolation, communication difficulties, and emotional overload are among the main elements contributing to the increased risk of suicide in this population. This scenario reinforces the urgency of implementing comprehensive preventive strategies that integrate psychological support, pharmacological interventions, and initiatives focused on social inclusion, aiming to minimize the impact of comorbidities.

In the context of ADHD, pharmacological therapy, particularly the use of stimulants like methylphenidate, plays an essential role in managing core symptoms such as inattention and hyperactivity. Additionally, this type of intervention can bring complementary benefits by helping with emotional regulation, a difficulty often shared between individuals with ADHD and ASD [30].

Finally, the integrated management of ADHD, ASD, and psychiatric comorbidities requires a multidisciplinary approach combining medical interventions, psychotherapies, and educational support. In addition to improving clinical outcomes, strategies that promote social inclusion and address associated risks, such as suicide, are essential to enhance patients’ quality of life and emotional well-being [32].

## 5. Intellectual Disability

ID, previously referred to as mental retardation, is a condition that affects approximately 1–3% of the global population, originating before the age of 18 [33]. ID is characterized by deficits in cognitive skills and adaptive functioning, impacting personal independence, social participation, and academic or professional performance [21]. It is defined by impairments in intellectual functioning (e.g., reasoning, problem-solving, and planning) and adaptive behavior (e.g., social, conceptual, and practical skills), creating challenges in personal independence and social responsibility [34].

**Figure 4:**
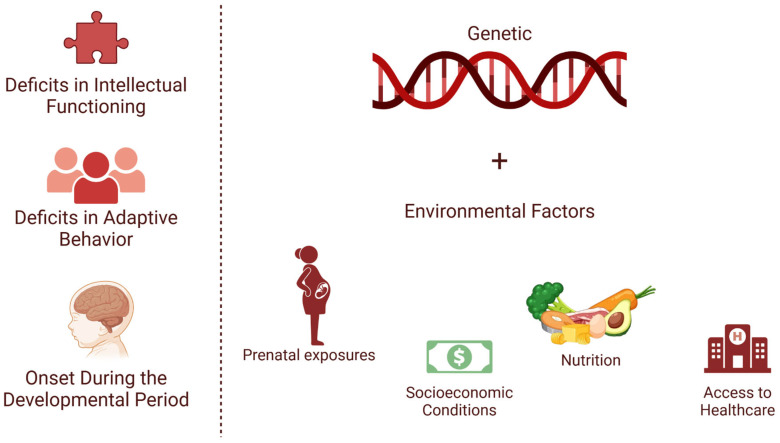
The core diagnostic criteria and etiological factors of intellectual disability (ID). ID diagnosis is based on three main criteria: deficits in intellectual functioning, deficits in adaptive behavior, and onset during the developmental period. The etiology of ID is multifactorial, encompassing genetic factors and environmental influences, such as prenatal exposures, socioeconomic conditions, nutrition, and access to healthcare.

The diagnosis of ID is guided by criteria outlined in the Diagnostic and Statistical Manual of Mental Disorders, Fifth Edition, Text Revision (DSM-5-TR). These criteria, as illustrated in Figure 4, emphasize three core elements: deficits in intellectual functioning, measured through standardized IQ tests, typically with scores below 70; deficits in adaptive behavior, evaluated across conceptual, social, and practical domains; and onset during the developmental period, distinguishing ID from acquired disabilities in adulthood [21].

The etiologies of ID are diverse and can be broadly categorized as genetic, environmental, and multifactorial. Genetic factors include chromosomal abnormalities, such as Down syndrome and Fragile X syndrome, and single-gene mutations, such as Rett syndrome, which are among the most studied genetic causes [35]. Recent advances in whole-genome sequencing have expanded the identification of pathogenic variants associated with ID [36]. Environmental factors include prenatal exposures, such as maternal infections (e.g., rubella, cytomegalovirus) and substance use (e.g., alcohol, drugs), as well as perinatal complications like hypoxia [37]. The interaction of genetic predisposition and environmental factors, including socioeconomic conditions, nutrition, and access to healthcare, also contribute to ID development [38]. Figure 4 provides a comprehensive overview of the multifactorial etiology of ID, highlighting the interplay between genetic and environmental factors.

Research has uncovered intriguing connections between ID and MDs, suggesting a potential neurobiological link between these conditions. Individuals with ID have a higher prevalence of MDs, such as depression and anxiety, compared to the general population [39,40]. For example, individuals with specific genetic syndromes associated with ID, such as Fragile X syndrome and Down syndrome, have an increased risk of developing MDs [41,42,43]. Neuroimaging studies have also revealed differences in brain structure and function in individuals with ID, which may contribute to their increased vulnerability to MDs [44].

Additionally, environmental factors, such as social isolation, limited access to resources, socioeconomic status, job satisfaction, and stressful life events, may compound the challenges faced by individuals with ID, further increasing their risk of developing MDs [45,46]. Biopsychosocial factors associated with depression and anxiety in older adults with ID include treatable and modifiable factors like mood stabilizer medications and aggressive challenging behavior [47].

## 6. Learning Disorders

**Figure 5:**
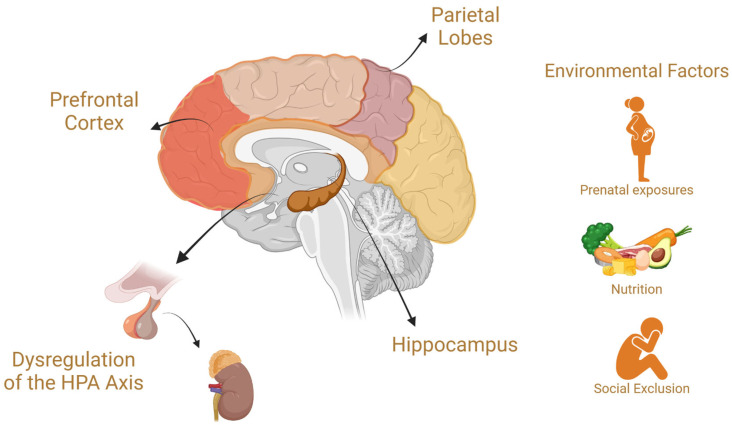
The neurological and environmental factors associated with learning disorders (LDs). The etiology of LDs is multifactorial, involving genetic, neurological, and environmental influences. Neurological differences in key brain regions, such as the prefrontal cortex, hippocampus, and parietal lobes, impact neural connectivity, processing speed, and executive functioning, which are critical for learning and memory. Additionally, dysregulation of the hypothalamic–pituitary–adrenal (HPA) axis has been implicated in LDs, linking stress-related neurobiological mechanisms to cognitive difficulties. Environmental factors, including prenatal exposures, nutritional deficiencies, and social exclusion, further contribute to LD risk through epigenetic and developmental pathways.

Figure 5 illustrates the key brain regions affected in learning disorders (LDs) and explores the influence of environmental factors.

Learning disorders (LDs) are neurodevelopmental disorders of biological origin, characterized by persistent difficulties in acquiring fundamental academic skills. These difficulties emerge during schooling and cannot be explained by other intellectual or sensory deficits [21]. Affecting 3% to 10% of children, LDs significantly impair the ability to acquire, retain, and apply information, impacting academic performance and everyday skills [48]. These conditions encompass a range of disorders, including dyslexia, dyscalculia, and ADHD, each with distinct but overlapping characteristics. Dyslexia, for instance, primarily impairs reading and language-related abilities, while dyscalculia affects mathematical reasoning and number comprehension [49]. ADHD, on the other hand, is characterized by difficulties in maintaining attention, regulating impulses, and managing hyperactivity, which can indirectly impact learning [50].

The etiology of LDs is complex and multifaceted, involving an interplay of genetic, neurological, and environmental factors. Studies have identified heritable components linked to specific genes that influence brain function and structure [51,52]. Neurological factors also contribute, with differences in brain regions such as the prefrontal cortex, hippocampus, and parietal lobes observed in individuals with LDs. These differences affect neural connectivity, processing speed, and executive functioning, which are essential for learning and memory [53,54].

Environmental influences, including prenatal exposure to toxins, nutritional deficiencies, and adverse childhood experiences, further compound the risk of developing LDs through epigenetic mechanisms [55]. Educational and social environments can mitigate or exacerbate individuals’ challenges with these conditions [56].

Increasing evidence points to a potential relationship between LDs and MDs, suggesting that the two may be linked at a neurobiological level. For example, individuals with LDs often experience co-occurring mental health challenges, including higher rates of depression and anxiety compared to the general population [57,58].

Recent research has identified specific neural networks and brain regions that are typically altered in individuals with LDs, such as changes in the activity and connectivity of brain areas involved in language processing, attention, and executive function [59]. Interestingly, similar neurobiological alterations have also been observed in individuals with MDs, suggesting that there may be overlapping neural pathways and mechanisms that contribute to both types of disorders [57,60].

One potential explanation for the relationship between LDs and MDs is the impact of academic and social challenges on an individual’s psychological well-being. Learning difficulties often lead to low self-esteem, frustration, and feelings of inadequacy, particularly in environments where academic performance is heavily emphasized [54]. Social exclusion is another contributing factor; children with ADHD or dyslexia, for instance, may experience bullying or isolation, which can heighten anxiety and depression. This social-emotional stress can further impair cognitive functioning, creating a vicious cycle that exacerbates both learning and MDs [61].

The interplay between stress and LDs is also explained by neurobiological mechanisms, particularly dysregulation of the HPA axis. Chronic stress associated with unmet academic expectations has been shown to impair hippocampal function, critical for memory and learning, and to increase susceptibility to MDs [62]. Furthermore, comorbid MDs, such as depression, can worsen cognitive impairments, creating significant challenges for academic achievement and overall quality of life [63].

## 7. Communication Disorders

Communication disorders (CDs), such as stuttering, speech sound disorders, aphasia, and social communication disorders, encompass a broad spectrum of difficulties in the development of language, speech, and social communication, in addition to childhood stuttering, affecting fluency and motor production of speech, with functional impacts throughout life [21,64]. These challenges can profoundly affect an individual’s ability to express themselves and engage in social interactions, often contributing to the development and manifestation of MDs, such as depression and anxiety [65].

The intricate relationship between CDs and MDs has been the subject of intense scientific scrutiny. Neuropsychiatry researchers have sought to unravel the complex neurobiological mechanisms that underlie the compelling connections between these two critical domains of human functioning [66].

Research suggests that the neurobiological mechanisms driving this connection involve processes related to language processing, social cognition, and emotional regulation. For instance, individuals with aphasia may experience frustration and isolation due to their inability to communicate effectively, which can increase their risk of depression [67]. Similarly, children with social communication disorder often face difficulties in peer relationships, which may lead to anxiety over time [68].

A study has highlighted the significant role of synaptic plasticity in the pathogenesis of MDs. Disruptions in the functional and structural connections of the neural circuits that underlie mood regulation have been implicated as a key driver of depressive symptomatology. Various risk factors, including stress-induced physiological changes, can cause these disruptions. Communication disorders have also been linked to alterations in neural connectivity and synaptic function, suggesting a potential shared neurobiological basis between these two conditions [66].

The neurobiology of severe mood and anxiety disorders has been extensively studied, revealing the involvement of multiple neurotransmitter systems and neural networks in regulating mood, emotion, and behavior. The frontal lobe, particularly the prefrontal cortex, has been identified as a key region in these processes. Responsible for executive functions such as decision-making, impulse control, and social behavior modulation, the prefrontal cortex is often impaired in individuals with CDs, which frequently co-occur with mood and anxiety disorders [64].

In addition to these mechanisms, recent studies have pointed to the role of cortical excitability and dopaminergic pathways in the relationship between communication disorders and MDs. Altered excitability, particularly in the motor cortex and language areas, may contribute to speech production and perception difficulties, exacerbating emotional distress and frustration. Furthermore, the dopaminergic system, important in motivation and reward processing, is dysregulated in both CDs and MDs. This dysregulation may impair the individual’s ability to experience positive reinforcement, thus reinforcing feelings of helplessness and anxiety [69,70].

Moreover, emerging research has identified inflammatory processes as an essential factor in the pathophysiology of both MDs and communication difficulties. Chronic inflammation has been implicated in the dysregulation of neurotransmitter systems, such as serotonin and glutamate, which are critical for mood regulation and cognitive functions like language processing. Elevated levels of inflammatory cytokines may disrupt neural communication, particularly in brain regions responsible for speech and emotional regulation [71,72,73].

**Figure 6:**
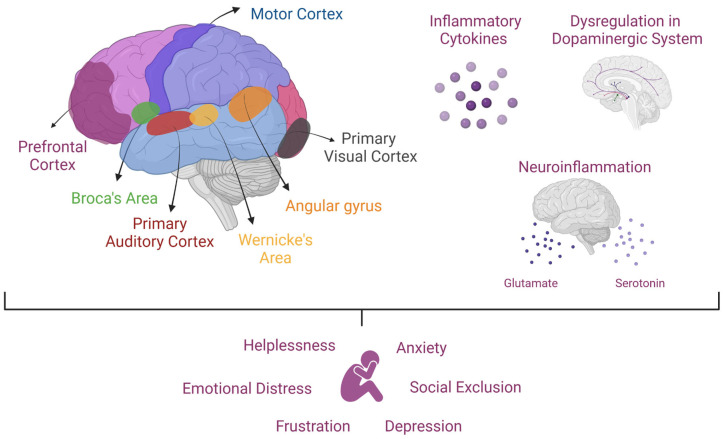
The neurological, neurochemical, and emotional impacts of communication disorders (CDs). CDs are associated with structural and functional alterations in key brain regions, including the prefrontal cortex, motor cortex, and language areas (Broca’s area, Wernicke’s area, primary auditory cortex, primary visual cortex, and the angular gyrus) which are essential for language processing and executive functions. Dysregulation in dopaminergic pathways, cortical excitability, and neuroinflammation—mediated by inflammatory cytokines and disruptions in serotonin and glutamate signaling—further contribute to language and communication difficulties. The cumulative effect of these neurological and biochemical disruptions often manifests as emotional distress, anxiety, frustration, helplessness, and depression, exacerbated by social exclusion and impaired peer interactions.

Figure 6 visually integrates these interconnected factors, highlighting how neurological, neurochemical, and environmental influences contribute to the emotional challenges associated with CDs.

## 8. Diagnostic and Therapeutic Implications

Advances in diagnostic methodologies have significantly enhanced our understanding of NDDs and MDs. Innovative genetic techniques, such as next-generation sequencing (NGS), have emerged as pivotal tools for identifying genetic variants associated with NDDs and improving diagnostic precision. For instance, studies using targeted gene panels and exome sequencing have demonstrated diagnostic yields of up to 25% in patients with syndromic or nonsyndromic NDDs, underscoring the importance of these technologies in personalized medicine [74,75]. These methods also facilitate early detection, enabling timely therapeutic interventions and better management outcomes.

Integrating neuroimaging technologies, such as functional magnetic resonance imaging (fMRI), has further elucidated the shared neural pathways involved in NDDs and MDs. Research highlights the role of altered connectivity in regions like the prefrontal cortex and amygdala in both conditions, paving the way for novel therapeutic approaches targeting these networks [76]. Moreover, diffusion tensor imaging (DTI) has shed light on white matter abnormalities in NDDs and MDs, emphasizing the importance of understanding brain network dynamics to develop precision diagnostics [77].

## 9. Pharmacological Strategies for Managing NDDs

In the therapeutic domain, advancements in pharmacological strategies include medications targeting specific neurotransmitter systems. For example, aripiprazole and risperidone have been shown to alleviate symptoms of irritability and aggression in children with ASD [78]. Moreover, selective serotonin reuptake inhibitors (SSRIs) and dopamine receptor modulators are increasingly employed to address overlapping symptoms in MDs and NDDs, demonstrating efficacy in both conditions when appropriately tailored [8]. Research into glutamatergic systems and their modulation through agents like memantine further illustrates the expanding scope of pharmacological interventions in treating complex NDD cases [79].

## 10. Behavioral Strategies for Managing NDDs

On the behavioral front, evidence-based interventions remain essential for improving quality of life and reducing the burden of NDD symptoms. Cognitive behavioral therapy (CBT) is widely employed, particularly for managing co-occurring anxiety and depression in individuals with NDDs. CBT focuses on modifying negative thought patterns and behaviors, improving emotional regulation, and promoting adaptive coping strategies. Applied behavior analysis (ABA) continues to be a cornerstone in addressing social, communication, and behavioral deficits in individuals with ASD. ABA utilizes reinforcement strategies to increase desirable behaviors while reducing maladaptive ones [80].

Additionally, newer interventions incorporating digital health tools—such as mobile applications and wearable devices—are increasingly integrated into therapy regimens. These tools track patient progress, offer real-time feedback, and help personalize interventions to better suit individual needs, further enhancing treatment outcomes [81].

## 11. Personalized Treatment Plans and Barriers to Implementation

Personalized treatment plans informed by genetic and environmental risk profiles are becoming central to targeted interventions. For example, variations in the CRHR1 gene associated with stress response dysregulation have guided therapies addressing MDs linked to early life trauma [12]. Similarly, pharmacogenomic approaches have helped optimize drug responses, reducing adverse effects and enhancing treatment efficacy in complex cases [82].

Despite these advancements, significant gaps remain in translating diagnostic insights into clinical practice. Barriers such as delayed diagnoses, lack of access to genetic counseling, and socioeconomic disparities hinder the practical application of these advancements [83]. Furthermore, the lack of integration across disciplines like psychiatry, neurology, and genetics often results in fragmented care, underscoring the need for more cohesive diagnostic and therapeutic strategies [84].

Family-based interventions and community support systems have also gained prominence in therapeutic frameworks. Studies suggest that empowering caregivers through psychoeducation and resource access significantly enhances treatment adherence and outcomes [85]. Moreover, policy-level changes, such as improving healthcare access and funding for genetic testing, remain essential for bridging gaps in care and ensuring equitable access to these advanced diagnostic and therapeutic options.

## 12. Final Considerations

The relationship between ID, LDs, and CDs and MDs highlights complex interactions that contribute to the challenges individuals face in both cognitive and emotional domains.

Research has shown that individuals with ID exhibit higher rates of MDs, such as depression and anxiety, compared to the general population [80]. Genetic factors, such as chromosomal abnormalities and gene mutations, along with environmental influences like prenatal exposure to infections or substances, contribute to the development of ID. Neuroimaging studies further support the theory that structural and functional brain differences in individuals with ID might predispose them to MDs. Moreover, social factors such as isolation and lack of resources may amplify the risks of developing MDs in individuals with ID, creating a cycle of exacerbating cognitive and emotional difficulties.

Similarly, LDs, including dyslexia, ADHD, and dyscalculia, are linked with MDs due to neurobiological alterations that affect brain areas involved in attention, language processing, and executive function. These disorders share overlapping neural pathways with MDs, which are often further complicated by the social–emotional stress individuals experience. Academic struggles and the frustration stemming from them can significantly impact self-esteem, potentially leading to depression and anxiety. Additionally, chronic stress has been shown to affect hippocampal function, thereby impairing cognitive performance and increasing susceptibility to MDs. Both ASD and ADHD are also frequently associated with mood disturbances, mainly due to the challenges in social interactions, emotional regulation, and executive functioning that these individuals face.

In communication disorders, such as aphasia and social communication disorder, the inability to effectively communicate can lead to frustration, social isolation, and emotional distress, which are significant risk factors for depression and anxiety. The neurobiological mechanisms underlying these disorders involve alterations in neural connectivity and synaptic plasticity, which are also implicated in mood regulation. Recent studies have highlighted the role of neurotransmitter systems, such as the dopaminergic system, in the overlap between mood and communication disorders, with dysregulation potentially leading to impaired emotional processing and the exacerbation of feelings of helplessness [86]. Moreover, inflammatory processes implicated in MDs and communication difficulties may disrupt neurotransmitter systems critical for mood and language processing, suggesting a shared pathophysiological basis for these disorders.

Some neurobiological features common to NDDs and MDs relate to synaptic structure and impairments in local connections and between brain regions involved in MDs and NDDs. These biological processes and mechanisms often underlie genetic polymorphisms or mutations, which direct changes in proteins involved in synaptic structure and connection.

An example of a gene that appears to have structural and functional involvement in circuits related to MDs and NDDs is the protocadherin 17 gene (PCDH17), which encodes a protein of the cadherin family. A study observed a relationship between the PCDH17 and MDs, cognition, and personality. A single-nucleotide polymorphism (SNP) of PCDH17, related to increased transcription, was associated with changes in morphology and reduced density of dendritic spines, reduced amygdala volume, increased MD prevalence, cognitive impairments, and emotional instability [87].

As previously reported, knowledge of genetic characteristics, such as polymorphisms, in addition to the local interaction between underlying brain structures in both NDDs and MDs, is crucial for advancing the understanding of these disorders and, consequently, for proposing and discovering more effective and common therapeutic strategies for them.

From another angle, the complex interactions between NDDs and MDs highlight the necessity of an integrated approach to treatment. Considering both these conditions’ cognitive and emotional dimensions is essential to provide a holistic and effective intervention. By addressing the underlying neurobiological, social, and emotional factors, individuals can have better support in navigating these challenges, improving their overall well-being and quality of life.

## Data Availability

Data sharing is not applicable to this article as no new data were created or analyzed in this study.

## References

[1] Yang Y., Zhao S., Zhang M., Xiang M., Zhao J., Chen S., Wang H., Han L., Ran J. (2022). Prevalence of Neurodevelopmental Disorders among US Children and Adolescents in 2019 and 2020. Front. Psychol..

[2] Bădescu G.M., Fîlfan M., Sandu R.E., Surugiu R., Ciobanu O., Popa-Wagner A. (2016). Molecular Mechanisms Underlying Neurodevelopmental Disorders, ADHD and Autism. Rom. J. Morphol. Embryol. Rev. Roum. Morphol. Embryol..

[3] Gillentine M.A., Wang T., Eichler E.E. (2022). Estimating the Prevalence of De Novo Monogenic Neurodevelopmental Disorders from Large Cohort Studies. Biomedicines.

[4] Steel Z., Marnane C., Iranpour C., Chey T., Jackson J.W., Patel V., Silove D. (2014). The Global Prevalence of Common Mental Disorders: A Systematic Review and Meta-Analysis 1980–2013. Int. J. Epidemiol..

[5] Halvorsen M., Mathiassen B., Myrbakk E., Brøndbo P.H., Sætrum A., Steinsvik O.O., Martinussen M. (2019). Neurodevelopmental Correlates of Behavioural and Emotional Problems in a Neuropaediatric Sample. Res. Dev. Disabil..

[6] Sesso G., Cristofani C., Berloffa S., Cristofani P., Fantozzi P., Inguaggiato E., Narzisi A., Pfanner C., Ricci F., Tacchi A. (2020). Autism Spectrum Disorder and Disruptive Behavior Disorders Comorbidities Delineate Clinical Phenotypes in Attention-Deficit Hyperactivity Disorder: Novel Insights from the Assessment of Psychopathological and Neuropsychological Profiles. J. Clin. Med..

[7] Safar K., Vandewouw M.M., Pang E.W., De Villa K., Crosbie J., Schachar R., Iaboni A., Georgiades S., Nicolson R., Kelley E. (2022). Shared and Distinct Patterns of Functional Connectivity to Emotional Faces in Autism Spectrum Disorder and Attention-Deficit/Hyperactivity Disorder Children. Front. Psychol..

[8] Wang T., Nowrangi D., Yu L., Lu T., Tang J., Han B., Ding Y., Fu F., Zhang J.H. (2018). Activation of Dopamine D1 Receptor Decreased NLRP3-Mediated Inflammation in Intracerebral Hemorrhage Mice. J. Neuroinflamm..

[9] Redenšek S., Blagus T., Trošt M., Dolžan V. (2022). Serotonin-Related Functional Genetic Variants Affect the Occurrence of Psychiatric and Motor Adverse Events of Dopaminergic Treatment in Parkinson’s Disease: A Retrospective Cohort Study. J. Pers. Med..

[10] Pizzagalli D.A., Berretta S., Wooten D., Goer F., Pilobello K.T., Kumar P., Murray L., Beltzer M., Boyer-Boiteau A., Alpert N. (2019). Assessment of Striatal Dopamine Transporter Binding in Individuals With Major Depressive Disorder: In Vivo Positron Emission Tomography and Postmortem Evidence. JAMA Psychiatry.

[11] Koenders M.A., Mesman E., Giltay E.J., Elzinga B.M., Hillegers M.H.J. (2020). Traumatic Experiences, Family Functioning, and Mood Disorder Development in Bipolar Offspring. Br. J. Clin. Psychol..

[12] Sanabrais-Jiménez M.A., Sotelo-Ramirez C.E., Ordoñez-Martinez B., Jiménez-Pavón J., Ahumada-Curiel G., Piana-Diaz S., Flores-Flores G., Flores-Ramos M., Jiménez-Anguiano A., Camarena B. (2019). Effect of CRHR1 and CRHR2 Gene Polymorphisms and Childhood Trauma in Suicide Attempt. J. Neural Transm..

[13] Vester A.I., Hermetz K., Burt A., Everson T., Marsit C.J., Caudle W.M. (2020). Combined Neurodevelopmental Exposure to Deltamethrin and Corticosterone Is Associated with Nr3c1 Hypermethylation in the Midbrain of Male Mice. Neurotoxicol. Teratol..

[14] Straub L., Bateman B.T., Hernandez-Diaz S., York C., Lester B., Wisner K.L., McDougle C.J., Pennell P.B., Gray K.J., Zhu Y. (2022). Neurodevelopmental Disorders Among Publicly or Privately Insured Children in the United States. JAMA Psychiatry.

[15] Besterman A.D., Adams D.J., Wong N.R., Schneider B.N., Mehta S., DiStefano C., Wilson R.B., Martinez-Agosto J.A., Jeste S.S. (2024). Genomics-Informed Neuropsychiatric Care for Neurodevelopmental Disorders: Results from A Multidisciplinary Clinic. Anesth. Analg..

[16] Rai S., Griffiths K.R., Breukelaar I.A., Barreiros A.R., Boyce P., Hazell P., Foster S.L., Malhi G.S., Harris A.W.F., Korgaonkar M.S. (2022). Common and Differential Neural Mechanisms Underlying Mood Disorders. Bipolar Disord..

[17] Deng Q., Hu L., Ding Y.-Q., Lang B. (2023). Editorial: The Commonality in Converged Pathways and Mechanisms Underpinning Neurodevelopmental and Psychiatric Disorders. Front. Mol. Neurosci..

[18] Marcolongo-Pereira C., Castro F.C.D.A.Q., Barcelos R.M., Chiepe K.C.M.B., Rossoni Junior J.V., Ambrosio R.P., Chiarelli-Neto O., Pesarico A.P. (2022). Neurobiological Mechanisms of Mood Disorders: Stress Vulnerability and Resilience. Front. Behav. Neurosci..

[19] De Marco R.L., Daniel M.B.N., Calvo E.N., Araldi B.L. (2021). Tea e Neuroplasticidade: Identificação e Intervenção Precoce/Asd and Neuroplasticity: Identification and Early Intervention. Braz. J. Dev..

[20] Wittkopf S., Stroth S., Langmann A., Wolff N., Roessner V., Roepke S., Poustka L., Kamp-Becker I. (2022). Differentiation of Autism Spectrum Disorder and Mood or Anxiety Disorder. Autism.

[21] American Psychiatric Association Publishing (2017). Diagnostic and Statistical Manual of Mental Disorders: DSM-5.

[22] Lord C., Brugha T.S., Charman T., Cusack J., Dumas G., Frazier T., Jones E.J.H., Jones R.M., Pickles A., State M.W. (2020). Autism Spectrum Disorder. Nat. Rev. Dis. Primer.

[23] Portolese J., Gomes C.S., Daguano Gastaldi V., Paula C.S., Caetano S.C., Bordini D., Brunoni D., Mari J.D.J., Vêncio R.Z.N., Brentani H. (2024). A Normative Model Representing Autistic Individuals Amidst Autism Spectrum Phenotypic Heterogeneity. Brain Sci..

[24] Morie K.P., Jackson S., Zhai Z.W., Potenza M.N., Dritschel B. (2019). Mood Disorders in High-Functioning Autism: The Importance of Alexithymia and Emotional Regulation. J. Autism Dev. Disord..

[25] Sanders S.J., He X., Willsey A.J., Ercan-Sencicek A.G., Samocha K.E., Cicek A.E., Murtha M.T., Bal V.H., Bishop S.L., Dong S. (2015). Insights into Autism Spectrum Disorder Genomic Architecture and Biology from 71 Risk Loci. Neuron.

[26] Vinci M., Treccarichi S., Galati Rando R., Musumeci A., Todaro V., Federico C., Saccone S., Elia M., Calì F. (2024). A de Novo ARIH2 Gene Mutation Was Detected in a Patient with Autism Spectrum Disorders and Intellectual Disability. Sci. Rep..

[27] Casellas-Vidal D., Mademont-Soler I., Sánchez J., Plaja A., Castells N., Camós M., Nieto-Moragas J., Del Mar García M., Rodriguez-Solera C., Rivera H. (2023). *ZDHHC15* as a Candidate Gene for Autism Spectrum Disorder. Am. J. Med. Genet. A.

[28] Palacios-Muñoz A., De Paula Moreira D., Silva V., García I.E., Aboitiz F., Zarrei M., Campos G., Rennie O., Howe J.L., Anagnostou E. (2022). Mutations in Trpγ, the Homologue of TRPC6 Autism Candidate Gene, Causes Autism-like Behavioral Deficits in Drosophila. Mol. Psychiatry.

[29] Hahamy A., Behrmann M., Malach R. (2015). The Idiosyncratic Brain: Distortion of Spontaneous Connectivity Patterns in Autism Spectrum Disorder. Nat. Neurosci..

[30] Oakley B., Loth E., Murphy D.G. (2021). Autism and Mood Disorders. Int. Rev. Psychiatry.

[31] Walton E., Pingault J.-B., Cecil C.A.M., Gaunt T.R., Relton C.L., Mill J., Barker E.D. (2017). Epigenetic Profiling of ADHD Symptoms Trajectories: A Prospective, Methylome-Wide Study. Mol. Psychiatry.

[32] Ruggieri V. (2020). Autism, depression and risk of suicide. Medicina.

[33] Olusanya B.O., Smythe T., Ogbo F.A., Nair M.K.C., Scher M., Davis A.C. (2023). Global Prevalence of Developmental Disabilities in Children and Adolescents: A Systematic Umbrella Review. Front. Public Health.

[34] Sappok T., Hassiotis A., Bertelli M., Dziobek I., Sterkenburg P. (2022). Developmental Delays in Socio-Emotional Brain Functions in Persons with an Intellectual Disability: Impact on Treatment and Support. Int. J. Environ. Res. Public Health.

[35] Ilyas M., Mir A., Efthymiou S., Houlden H. (2020). The Genetics of Intellectual Disability: Advancing Technology and Gene Editing. F1000Research.

[36] Lindstrand A., Eisfeldt J., Pettersson M., Carvalho C.M.B., Kvarnung M., Grigelioniene G., Anderlid B.-M., Bjerin O., Gustavsson P., Hammarsjö A. (2019). From Cytogenetics to Cytogenomics: Whole-Genome Sequencing as a First-Line Test Comprehensively Captures the Diverse Spectrum of Disease-Causing Genetic Variation Underlying Intellectual Disability. Genome Med..

[37] Leonard H., Montgomery A., Wolff B., Strumpher E., Masi A., Woolfenden S., Williams K., Eapen V., Finlay-Jones A., Whitehouse A. (2022). A Systematic Review of the Biological, Social, and Environmental Determinants of Intellectual Disability in Children and Adolescents. Front. Psychiatry.

[38] Tchoua P.P., Clarke E., Wasser H., Agrawal S., Scothorn R., Thompson K., Schenkelberg M., Willis E.A. (2025). The Interaction between Social Determinants of Health, Health Behaviors, and a Child’s Intellectual and Developmental Diagnoses. Transl. J. Am. Coll. Sports Med..

[39] Eaton C., Tarver J., Shirazi A., Pearson E., Walker L., Bird M., Oliver C., Waite J. (2021). A Systematic Review of the Behaviours Associated with Depression in People with Severe–Profound Intellectual Disability. J. Intellect. Disabil. Res..

[40] Albuquerque J.P. (2023). Diagnostic Issues in Other Mental Disorders Co-Morbid With Intellectual Disability. Eur. Psychiatry.

[41] Adams R.L., Baird A., Smith J., Williams N., Van Den Bree M.B.M., Linden D.E.J., Owen M.J., Hall J., Linden S.C. (2023). Psychopathology in Adults with Copy Number Variants. Psychol. Med..

[42] Lightbody A.A., Bartholomay K.L., Jordan T.L., Lee C.H., Miller J.G., Reiss A.L. (2022). Anxiety, Depression, and Social Skills in Girls with Fragile X Syndrome: Understanding the Cycle to Improve Outcomes. J. Dev. Behav. Pediatr..

[43] Rivelli A., Fitzpatrick V., Chaudhari S., Chicoine L., Jia G., Rzhetsky A., Chicoine B. (2022). Prevalence of Mental Health Conditions Among 6078 Individuals With Down Syndrome in the United States. J. Patient-Cent. Res. Rev..

[44] Barua P.D., Vicnesh J., Gururajan R., Oh S.L., Palmer E., Azizan M.M., Kadri N.A., Acharya U.R. (2022). Artificial Intelligence Enabled Personalised Assistive Tools to Enhance Education of Children with Neurodevelopmental Disorders—A Review. Int. J. Environ. Res. Public Health.

[45] Clark L.M., Kelley M.L., Matson J.L. (2020). Risk Factors for Dual Disorders in Individuals with Intellectual Disabilities. Handbook of Dual Diagnosis.

[46] Dow M., Long B., Lund B. (2021). Reference and Instructional Services to Postsecondary Education Students with Intellectual Disabilities. Coll. Res. Libr..

[47] Bosch O.J., Nair H.P., Ahern T.H., Neumann I.D., Young L.J. (2009). The CRF System Mediates Increased Passive Stress-Coping Behavior Following the Loss of a Bonded Partner in a Monogamous Rodent. Neuropsychopharmacology.

[48] Gupta N., Goyal N., Sharma E. (2022). Learning Disability Certification in India: Quo Vadis. J. Indian Assoc. Child Adolesc. Ment. Health.

[49] López-Resa P., Moraleda-Sepúlveda E. (2023). Working Memory Capacity and Text Comprehension Performance in Children with Dyslexia and Dyscalculia: A Pilot Study. Front. Psychol..

[50] Español-Martín G., Pagerols M., Prat R., Rivas C., Ramos-Quiroga J.A., Casas M., Bosch R. (2023). The Impact of Attention-Deficit/Hyperactivity Disorder and Specific Learning Disorders on Academic Performance in Spanish Children from a Low-Middle- and a High-Income Population. Front. Psychiatry.

[51] Higazi A.M., Kamel H.M., Abdel-Naeem E.A., Abdullah N.M., Mahrous D.M., Osman A.M. (2021). Expression Analysis of Selected Genes Involved in Tryptophan Metabolic Pathways in Egyptian Children with Autism Spectrum Disorder and Learning Disabilities. Sci. Rep..

[52] Kubota T., Misciagna S. (2022). Biological Understanding of Neurodevelopmental Disorders Based on Epigenetics, a New Genetic Concept in Education. Learning Disabilities—Neurobiology, Assessment, Clinical Features and Treatments.

[53] Avigan P.D., Cammack K., Shapiro M.L. (2020). Flexible Spatial Learning Requires Both the Dorsal and Ventral Hippocampus and Their Functional Interactions with the Prefrontal Cortex. Hippocampus.

[54] Qu J., Hu L., Liu X., Dong J., Yang R., Mei L. (2021). The Contributions of the Left Hippocampus and Bilateral Inferior Parietal Lobule to Form-meaning Associative Learning. Psychophysiology.

[55] Kundakovic M., Jaric I. (2017). The Epigenetic Link between Prenatal Adverse Environments and Neurodevelopmental Disorders. Genes.

[56] Ugalde L., Santiago-Garabieta M., Villarejo-Carballido B., Puigvert L. (2021). Impact of Interactive Learning Environments on Learning and Cognitive Development of Children With Special Educational Needs: A Literature Review. Front. Psychol..

[57] Kokot S.J. (2005). A Neurodevelopmental Approach for Helping Gifted Learners with Diagnosed Dyslexia and Attention Deficit/Hyperactivity Disorder (AD/HD). Afr. Educ. Rev..

[58] Colomer C., Berenguer C., Roselló B., Baixauli I., Miranda A. (2017). The Impact of Inattention, Hyperactivity/Impulsivity Symptoms, and Executive Functions on Learning Behaviors of Children with ADHD. Front. Psychol..

[59] Moreau D., Waldie K.E. (2016). Developmental Learning Disorders: From Generic Interventions to Individualized Remediation. Front. Psychol..

[60] Mohammed A.M., Bahar H.F., Karbala Governorate Education Directorate Ministry of Education (2024). Iraq Mood Disorder in Students with Learning Disabilities. Am. J. Soc. Sci. Educ. Innov..

[61] McEwen B.S., Gianaros P.J. (2011). Stress- and Allostasis-Induced Brain Plasticity. Annu. Rev. Med..

[62] Lupien S.J., McEwen B.S., Gunnar M.R., Heim C. (2009). Effects of Stress throughout the Lifespan on the Brain, Behaviour and Cognition. Nat. Rev. Neurosci..

[63] Maksyutynska K., Stogios N., Prasad F., Gill J., Hamza Z., De R., Smith E., Horta A., Goldstein B.I., Korczak D. (2024). Neurocognitive Correlates of Metabolic Dysregulation in Individuals with Mood Disorders: A Systematic Review and Meta-Analysis. Psychol. Med..

[64] Cai H., Dong J., Mei L., Feng G., Li L., Wang G., Yan H. (2024). Functional and Structural Abnormalities of the Speech Disorders: A Multimodal Activation Likelihood Estimation Meta-Analysis. Cereb. Cortex.

[65] Pascoe M.I., Forbes K., De La Roche L., Derby B., Psaradellis E., Anagnostou E., Nicolson R., Georgiades S., Kelley E. (2023). Exploring the Association between Social Skills Struggles and Social Communication Difficulties and Depression in Youth with Autism Spectrum Disorder. Autism Res..

[66] Mann J.J., Currier D., Quiroz J.A., Manji H.K. (2012). Neurobiology of Severe Mood and Anxiety Disorders. Basic Neurochemistry.

[67] Kao S.-K., Chan C.-T. (2024). Increased Risk of Depression and Associated Symptoms in Poststroke Aphasia. Sci. Rep..

[68] Wren Y., Pagnamenta E., Orchard F., Peters T.J., Emond A., Northstone K., Miller L.L., Roulstone S. (2023). Social, Emotional and Behavioural Difficulties Associated with Persistent Speech Disorder in Children: A Prospective Population Study. JCPP Adv..

[69] Fries G.R., Saldana V.A., Finnstein J., Rein T. (2023). Molecular Pathways of Major Depressive Disorder Converge on the Synapse. Mol. Psychiatry.

[70] Li Y., Zhao Y., Lu Y., Lu X., Hu Y., Li Q., Shuai M., Li R. (2022). Autism Spectrum Disorder-like Behavior Induced in Rat Offspring by Perinatal Exposure to Di-(2-Ethylhexyl) Phthalate. Environ. Sci. Pollut. Res..

[71] Poletti S., Mazza M.G., Benedetti F. (2024). Inflammatory Mediators in Major Depression and Bipolar Disorder. Transl. Psychiatry.

[72] Dall M., Fellinger J., Holzinger D. (2022). The Link between Social Communication and Mental Health from Childhood to Young Adulthood: A Systematic Review. Front. Psychiatry.

[73] Cantwell D.P. (1977). Psychiatric Disorder in Children With Speech and Language Retardation: A Critical Review. Arch. Gen. Psychiatry.

[74] Mellone S., Puricelli C., Vurchio D., Ronzani S., Favini S., Maruzzi A., Peruzzi C., Papa A., Spano A., Sirchia F. (2022). The Usefulness of a Targeted Next Generation Sequencing Gene Panel in Providing Molecular Diagnosis to Patients with a Broad Spectrum of Neurodevelopmental Disorders. Front. Genet..

[75] Postma J.K., Harrison M., Kutcher S., Webster R.J., Cloutier M., Bourque D.K., Yu A.C., Carter M.T. (2024). The Diagnostic Yield of Genetic and Metabolic Investigations in Syndromic and Nonsyndromic Patients with Autism Spectrum Disorder, Global Developmental Delay, or Intellectual Disability from a Dedicated Neurodevelopmental Disorders Genetics Clinic. Am. J. Med. Genet. A.

[76] Dajani D.R., Burrows C.A., Odriozola P., Baez A., Nebel M.B., Mostofsky S.H., Uddin L.Q. (2019). Investigating Functional Brain Network Integrity Using a Traditional and Novel Diagnostic System for Neurodevelopmental Disorders. Neuroimage Clin..

[77] Mohajer B., Masoudi M., Ashrafi A., Mohammadi E., Bayani Ershadi A.S., Aarabi M.H., Uban K.A. (2019). Structural White Matter Alterations in Male Adults with High Functioning Autism Spectrum Disorder and Concurrent Depressive Symptoms; a Diffusion Tensor Imaging Study. J. Affect. Disord..

[78] Iffland M., Livingstone N., Jorgensen M., Hazell P., Gillies D. (2023). Pharmacological Intervention for Irritability, Aggression, and Self-Injury in Autism Spectrum Disorder (ASD). Cochrane Database Syst. Rev..

[79] Canitano R., Scandurra V. (2014). Glutamatergic Agents in Autism Spectrum Disorders: Current Trends. Res. Autism Spectr. Disord..

[80] Yu Q., Li E., Li L., Liang W. (2020). Efficacy of Interventions Based on Applied Behavior Analysis for Autism Spectrum Disorder: A Meta-Analysis. Psychiatry Investig..

[81] Arabian H., Abdulbaki Alshirbaji T., Schmid R., Wagner-Hartl V., Chase J.G., Moeller K. (2023). Harnessing Wearable Devices for Emotional Intelligence: Therapeutic Applications in Digital Health. Sensors.

[82] Gill P.S., Elchynski A.L., Porter-Gill P.A., Goodson B.G., Scott M.A., Lipinski D., Seay A., Kehn C., Balmakund T., Schaefer G.B. (2022). Multidisciplinary Consulting Team for Complicated Cases of Neurodevelopmental and Neurobehavioral Disorders: Assessing the Opportunities and Challenges of Integrating Pharmacogenomics into a Team Setting. J. Pers. Med..

[83] Simon J., Hyde C., Saravanapandian V., Wilson R., Distefano C., Besterman A., Jeste S. (2022). The Diagnostic Journey of Genetically Defined Neurodevelopmental Disorders. J. Neurodev. Disord..

[84] Finucane B.M., Ledbetter D.H., Vorstman J.A. (2021). Diagnostic Genetic Testing for Neurodevelopmental Psychiatric Disorders: Closing the Gap between Recommendation and Clinical Implementation. Curr. Opin. Genet. Dev..

[85] Loader S.J., Brouwers N., Burke L.M. (2019). Neurodevelopmental Therapy Adherence in Australian Parent-Child Dyads: The Impact of Parental Stress. Educ. Dev. Psychol..

[86] Hamamah S., Aghazarian A., Nazaryan A., Hajnal A., Covasa M. (2022). Role of Microbiota-Gut-Brain Axis in Regulating Dopaminergic Signaling. Biomedicines.

[87] Chang H., Hoshina N., Zhang C., Ma Y., Cao H., Wang Y., Wu D., Bergen S.E., Landén M., Hultman C.M. (2018). The Protocadherin 17 Gene Affects Cognition, Personality, Amygdala Structure and Function, Synapse Development and Risk of Major Mood Disorders. Mol. Psychiatry.

